# Differential Effects of Ephemeral and Stable Predator Chemical Cues on Spider Antipredator Behaviour

**DOI:** 10.1007/s10886-024-01543-5

**Published:** 2024-09-21

**Authors:** Nijat Narimanov, Jonna M. Heuschele, Martin H. Entling, Florian Menzel, Laia Mestre

**Affiliations:** 1 iES Landau, Institute for Environmental Sciences, RPTU Kaiserslautern-Landau, 76829 Landau (Pfalz), Germany; 2 Institute of Organismic and Molecular Evolution (iomE), Johannes Gutenberg University of Mainz, 55128 Mainz, Germany; 3 Department of Community Ecology, UFZ, Helmholtz Centre for Environmental Research, 06120 Halle (Saale), Germany; 4 iDiv, German Centre for Integrative Biodiversity Research, Halle-Jena-Leipzig, 04103 Leipzig, Germany

**Keywords:** Araneae, Formicidae, Non-consumptive effects, *Pisaura mirabilis*, Semiochemicals, *Xysticus*

## Abstract

Semiochemicals left by predators in their foraging area can be utilised by prey to avoid predation. The range of predators’ chemical cues with contrasting degradation rates might provide information of different quality, potentially allowing prey to differentiate between the immediate and the longer-term presence of predators in a location. So far, knowledge about the roles of volatile versus stable chemical cues in informing predation risk is limited. We here seek to disentangle the role of ephemeral trail pheromones compared to persistent cuticular hydrocarbons of ants (predators) on the antipredator behaviour of juvenile spiders (prey), with the expectation that volatile semiochemicals induce avoidance behaviour in spiders at a higher rate compared to stable cues. We allowed the spiders to choose between sites with and without ant cues separately for volatile trail pheromones and stable hydrocarbons. Unexpectedly, spiders avoided the presence of persistent cuticular hydrocarbons more clearly than the highly volatile trail pheromone. This underscores the widespread impact of these stable cues on the avoidance behaviour of potential intraguild prey. The response to trail pheromones was unclear, possibly because spiders always encounter these cues simultaneously with visual and vibratory cues from ants; hence, trail pheromones may not contain any additional information, hindering the evolution of the ability to detect them.

**Supplementary Information:**

The online version contains supplementary material available at 10.1007/s10886-024-01543-5.

## Introduction

Apart from killing and consuming (consumptive effects), predators can also induce behavioural changes (non-consumptive effects) in their prey (Lima 1998; Werner and Peacor 2003). Such behavioural changes may result in activity and feeding rate changes that have an impact on lower trophic levels (Bucher et al. 2015b; Foam et al. 2005; Gazzola et al. 2015; Mestre et al. 2020; Peacor and Werner 2000; Thaler et al. 2012). Often, potential prey move away from sites where they detect cues of predators. While this can reduce mortality from predation, it can impose other fitness costs to the prey, such as giving up favourable foraging sites (Heiling and Herberstein 2004; Mestre et al. 2014; Morosinotto et al. 2010; Weisser et al. 1999). The consequences of such non-consumptive effects of predators on prey can be equivalent to or even stronger than the effects of direct mortality (Preisser et al. 2005; Peacor and Werner 2008). In most known examples, the predator avoidance behaviour in prey is based on chemical cues left by potential predators (e.g., Bucher et al. 2021; Geiselhardt et al. 2011; Mestre et al. 2014, 2020; Wüst and Menzel 2017). Despite the high importance, the chemical mechanisms underlying prey antipredator behaviours are still poorly understood. Further, experiments rarely address the identity and characteristics of the involved chemical cues (Buchanan et al. 2017), although detailed information about the chemicals would allow the design of more powerful studies and explain variability in the prey’s avoidance behaviour towards predators.

Chemical cues in the area can signal the presence of predators. However, they might also be largely uninformative regarding the specific/acute predation risk, for example, if they are uniformly present across large areas. Apart from external factors, several internal characteristics of predators’ semiochemicals might influence the quality of information provided by cues (for more details, see Table 1). Notably, specific chemical cues might not indicate the immediate presence of predation risk since cues with low degradation rates remain in the area relatively long after the presence of predators (Kats and Dill 1998; Smith and Belk 2001). For instance, the persistence of chemical cues of aquatic predators varies not only due to environmental factors (e.g., temperature) but also due to predator-intrinsic factors, such as the type of cues, which causes variability in the response of prey to predator presence (Van Buskirk et al. 2014). The persistence of the emitted chemical cues, hence, might play a more significant role in predator recognition by prey, with volatile cues informing on the more recent presence of predators in the area than relatively long-lived cues. On the other hand, long-lived cues stay detectable over longer periods and can, therefore, better inform about predators that repeatedly use their foraging grounds over longer times than short-lived cues. Consequently, prey might show diverse antipredator responses utilising differential signals provided by long- and short-lived chemical cues of predators. Nevertheless, evidence of the differential impact of predator chemical cues on prey behaviour, to our knowledge, is missing.

**Table 1:** List of several well-studied internal factors influencing the type of signal provided by predators’ chemical cues used by prey to detect predation risk

Factors	Context	Examples
Age	Avoidance behaviour decays with increasing cue age deposited in the area.	Coral reef damselfish (Chivers et al. 2013); Dragonfly (Peacor 2006); Gastropod (Turner and Montgomery 2003); Spider (Persons and Rypstra 2001); Wood frog tadpole (Ferrari et al. 2007).
Detectability	Higher detectability of cues left by predators with shared co-evolution history than by novel predators.	Ant (Bucher et al. 2021); Characid fish (Sharpe et al. 2021); Mussel (Freeman and Byers 2006).
Specificity	Cues also left by non-threatening organisms are less valuable in detecting predation risk than substances specific to dangerous species.	Atlantic salmon (Hawkins et al. 2007); Wood frog tadpole (Relyea 2003).
Strength	Stronger cues (e.g., chemicals left by predators in high concentrations or larger predators) are more likely to be detected and used than more subtle cues.	Atlantic salmon (Hawkins et al. 2007); Fathead minnow (Ferrari et al. 2006; Kusch et al. 2004); Spider (Barnes et al. 2002); Wood frog tadpole (Relyea 2003).
Predator diet	Cues released by predators fed on conspecific vs. heterospecific prey may trigger different strengths of avoidance behaviour.	Crayfish (Beattie and Moore 2018); Gastropod (Turner 2008).

In this study, we tested whether chemical cues of ant predators with differential volatility induce different antipredator responses in spider prey. Both spiders and ants are highly abundant and play a key role in terrestrial food webs as generalist predators (Michalko et al. 2019; Nyffeler and Birkhofer 2017; Schultheiss et al. 2022). Ants are natural enemies of spiders and often compete with them for food, strongly impacting spider assemblages (Mestre et al. 2013, 2016; Sanders et al. 2011). Furthermore, ants are also known as intraguild predators of spiders with the consequent negative effects on spider densities (Del-Claro and Oliveira 2000; Halaj et al. 1997; James et al. 1999; Seifert 2018). Spiders, therefore, avoid ant cues by increasing their dispersal propensity, as shown in experiments exposing spiders to footprints of the black garden ant *Lasius niger* (Mestre et al. 2014). Moreover, cursorial, but not sedentary, spider species increase their activity when exposed to the presence of ants, indicating their attempt to escape the risky area (Mestre et al. 2020). Ants use semiochemicals to communicate (Lenoir et al. 2001; Howard and Blomquist 2005), and the non-consumptive effects on other species likely arise when these eavesdrop on ant cues and use them as indicators of their presence (Binz et al. 2014; Mestre et al. 2014). The ant *Lasius niger* uses two main types of chemical signals to organise foraging decisions in the colony. On the one hand, worker ants that find a food source mark the pathway to the nest with trail pheromone until food becomes depleted (Beckers et al. 1992; David Morgan 2009). Trail pheromones are highly volatile, so an inactive trail becomes undetectable to ants within one hour (Beckers et al. 1993; Evison et al. 2008; Lenoir et al. 2009). On the other hand, workers passively leave hydrocarbons on the substrate as they walk, whose composition is very similar to cuticular hydrocarbons (Lenoir et al. 2009; Wüst and Menzel 2017). Since they are non-volatile, these footprint hydrocarbons serve as long-lasting home range markings that inform workers about the density and activity of nestmates at a given location, thereby promoting the exploitation of food sources to known sites (Devigne et al. 2004; Lenoir et al. 2001, 2009). Hydrocarbons and trail pheromones have contrasting decay rates. This allows ants to distinguish areas being visited at a given moment from recently visited areas and recruit accordingly (Czaczkes et al. 2012, 2015). This relationship between the temporal scale of cue degradation and the spatial scale of ant foraging activity may be exploited by other species to assess the risk of encountering ants.

We exposed two common grassland spiders (*Pisaura mirabilis*,* Xysticus* spp.) to stable cuticular hydrocarbons and volatile trail pheromones of the ant *L.niger*. We performed choice experiments and recorded the site-selection decisions of spiders at successive time points. We expected (1) that spiders prefer control sites over sites with chemical cues avoiding possible encounter with ants and (2) that cues with a high degradation rate (trail pheromone) induce stronger predator avoidance behaviour in spiders than stable cues (cuticular hydrocarbons) because the former indicates a higher level of threat (more immediate presence of predators). We also predicted (3) that spiders avoid cuticular hydrocarbons constantly over 24 h; however, only fresh trail pheromones would induce a similar response due to the ephemeral character of trail pheromones.

## Materials and Methods

### Study Organisms

We sampled our study organisms, namely juvenile spiders (*P. mirabilis* and *Xysticus* spp.) and ants (*L. niger*), between May and the beginning of July 2018 at two sites close to Landau (Rhineland-Palatinate, Germany). The first site, “Ebenberg”, is a former military area turned into a nature reserve with open dry grasslands grazed by sheep (49°10′53′′ N/8°7′52′′ E). The second site is a forest clearing with herbaceous and heath-like vegetation near the town of Bellheim (49°11′45′′ N/8°19′11′′ E). We used a vacuum sampler (modified STIHL SH86 blower; Stihl, Waiblingen, Germany) to collect small juvenile spiders from the herbaceous layer and *L. niger* workers at the entrance of their nests. *Lasius niger* occurs syntopically with *Xysticus* and *Pisaura* and, as an omnivorous species, often preys on them along with other arthropods (Seifert 2018). We could easily identify juvenile *P. mirabilis* to species in the field, whereas juvenile *Xysticus* spp. (thereafter *Xysticus*) were only identifiable by genus. Based on co-occurring adult individuals, *Xysticus* is probably comprised of a mix of *X. cristatus*, *X. kochi*, and *X. luctuosus*. *Pisaura mirabilis* spiders (Araneae: Pisauridae) build nursery webs for brood care, sheet webs as juveniles and are free hunters during later instars and as adults. *Xysticus* spp. (Araneae: Thomisidae) are ambush hunters (Cardoso et al. 2011; Lenler-Eriksen 1969; Nyffeler & Breene 1990; Pekar et al. 2021). Consequently, both families mainly use the sit-and-wait strategy for hunting, especially at the juvenile life stage. In the lab, we froze ant workers at -20 °C until we used them to extract ant cues. We kept spiders individually inside a climate cabinet (16 °C, RH = 65%, L: D = 12:12) in 3.5 cm-diameter Petri dishes. We fed spiders *ad libitum* with springtails (*Sinella curviseta*) and supplied moisture by placing a water-soaked cotton ball into the Petri dish. We measured each individual’s body length, prosoma width and weight to the nearest 0.01 mm and 0.01 mg, respectively. The spider’s prosoma width is widely used to proxy body size since it is independent of body conditions (Moya-Laraño et al. 2008).

### Extraction of Ant Cues

**Fig. 1:**
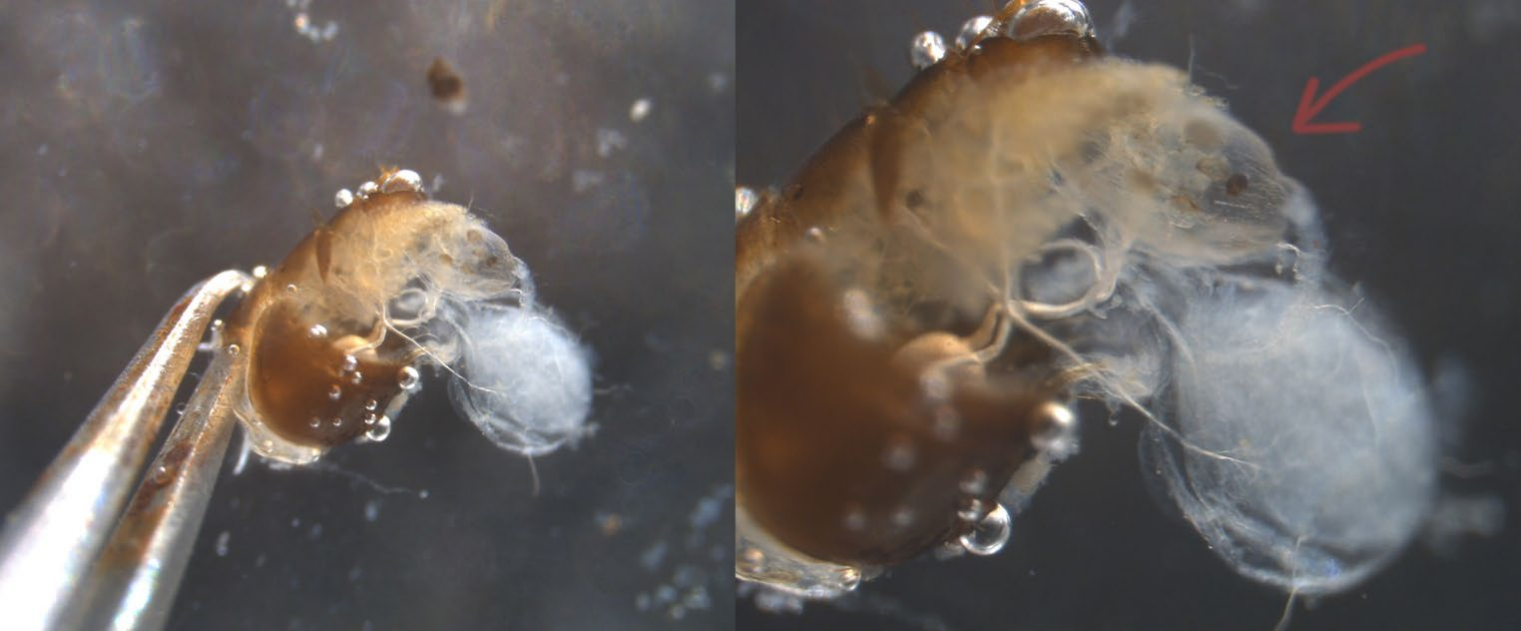
Abdominal dissection of the ant *Lasius niger* with the extraction of the hindgut gland containing trail pheromone. The hindgut gland is indicated by the arrow on the right photo. Dissection pictures were taken by FM and NN

To create cuticular hydrocarbon (CHC) stocks, we introduced 36 ant workers (killed by freezing) in 4 mL vials and pipetted 2 mL of *n*-hexane (C_6_H_14_) as a solvent to extract the cues from the workers’ cuticle. Ant cuticular hydrocarbons are complex mixtures of long-chained alkanes and alkenes with chain lengths between C20 and C40 (Sprenger and Menzel 2020). CHCs of *Lasius niger* contain mostly *n*-, monomethyl-, dimethyl-, and trimethyl- alkanes, as well as a small amount of alkenes (Lenoir et al. 2009; Wittke et al. 2022). All cuticular hydrocarbons are non-polar and, hence, dissolve best in non-polar solvents, which is why we used hexane to extract them from the ant cuticles. We removed the workers after 10 min and let the hexane evaporate over 24 h. We then pipetted 475 µL of hexane into the vials. To create stocks of trail pheromone (TP), we dissected the ants’ abdomen to extract the hindgut gland (Fig. 1), where the TP is produced (David Morgan 2009). We introduced 80 hindgut glands into 4 mL vials and filled them to the top with dichloromethane (CH_2_Cl_2_) as a solvent. The trail pheromone of *L. niger* consists of 3,4-Dihydro-8-hydroxy-3,5,7-trimethylisocoumarin (C_12_H_14_O_3_; Bestmann et al. 1992), which is a polar compound. Since polar compounds dissolve best in polar solvents, we used dichloromethane – a polar solvent – to prepare the trail pheromone solutions. To check whether the TP solution contained trail pheromone, we tested 30 workers on a Y-maze where we pipetted a trail of TP solution on one arm and dichloromethane alone on the other (Fig. S2 and S3 in supplementary materials). Around 90% of the workers followed the arm with the artificial TP solution, matching the path choice accuracy of natural trail pheromones in *L. niger* (Czaczkes et al. 2013, 2019; von Thienen et al. 2014). However, the ants could follow the correct path only for the first ~ 45 min after the trail pheromone solution application (NN & LM personal observation; Fig. S2 and S3 in supplementary materials), indicating once again a highly ephemeral character of trail pheromones. For a realistic dosage of our CHC solution, we adjusted the amount per treatment to the amount of CHC that is left by ten *L. niger* workers after walking for six hours (Wüst and Menzel 2017). We froze the stocks of chemical cues at -20 °C immediately after preparation until needed and always handled the solutions on ice during application.

### Experimental Design

**Fig. 2:**
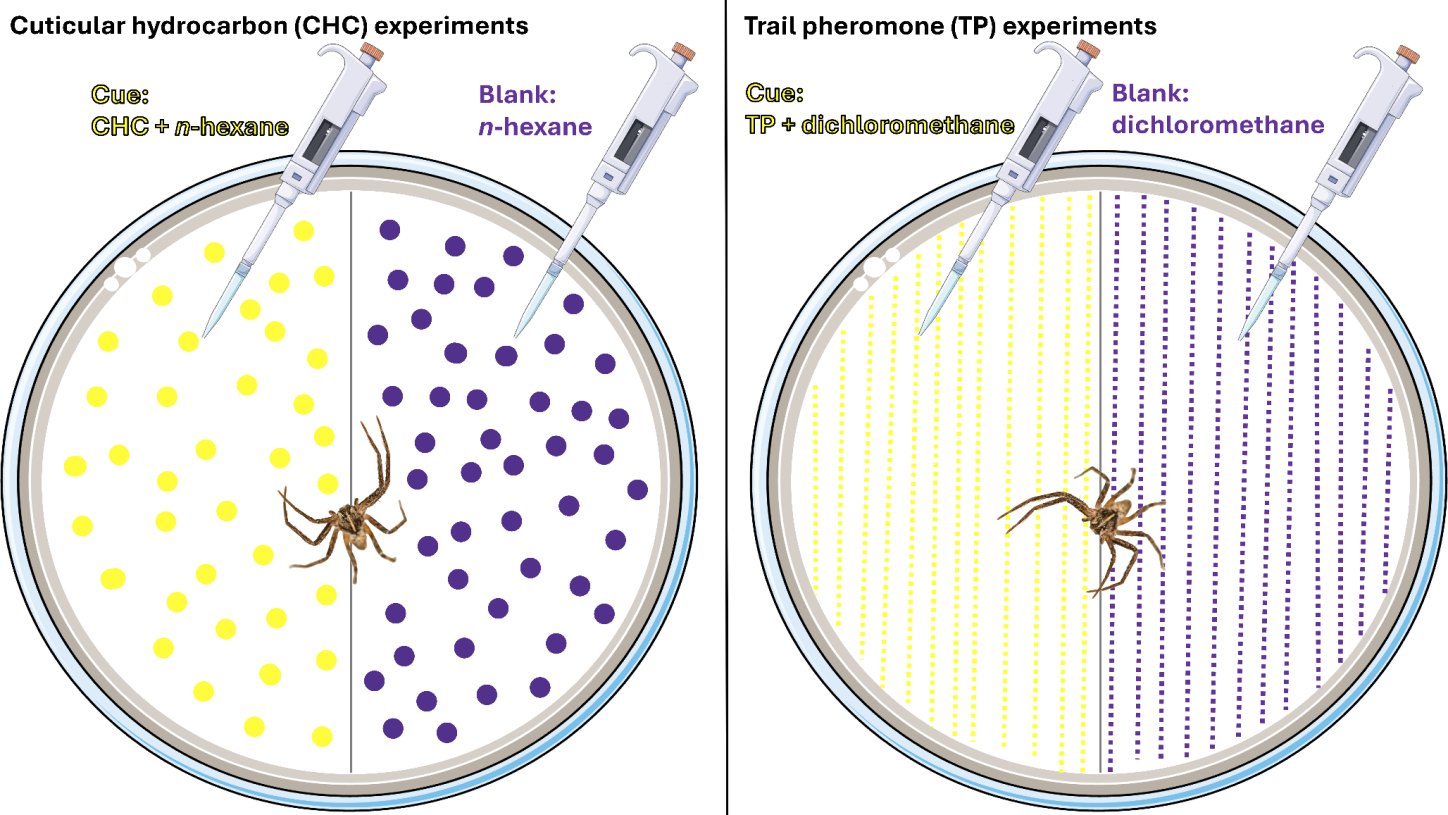
Experimental design for the cuticular hydrocarbon (CHC; left panel) and trail pheromone (TP; right panel). We applied the cue + solvent (CHC + *n*-hexane or TP + dichloromethane) on one side of the filter paper (cue treatment) and pure solvents (*n*-hexane or dichloromethane) on the other halves of each petri dish. Different sets of spiders were used for each experiment. ©: Petri-dish-top-gray icon and Micropipette icon by Servier https://smart.servier.com/ is licensed under CC-BY 3.0 Unported https://creativecommons.org/licenses/by/3.0/

To study how ant cues affect site selection, we placed spiders individually on a 9 cm-diameter filter paper, which was visually divided into two halves. Each half contained either ant cues (cue treatments) or no cues (blank; Fig. 2). To avoid creating a possible movement obstacle, we used the whole filter paper without cutting it into two halves. The choice experiments were conducted separately for each spider species and ant cue type (CHC, TP), using different sets of individuals (*n* = 48–54 per spider species and cue type, Table 2). We assigned individuals to either experiment randomly while ensuring that body size distribution did not differ between experiments to avoid potential confounding effects (Table 2). We could not determine the instar stage of all tested juvenile spiders since they were sampled from the fields. However, the tested spiders had similar body size parameters (Table 2), and no spider moulted during the 24 h of our experiments. On each half of the filter papers, we pipetted 36 µL of either solvent alone (= blank, *n*-hexane in the CHC experiment, dichloromethane in the TP experiment) or of actual cue solution. We pipetted the CHC solution in a dot-like fashion to mimic the ants’ walking pattern and the TP solution in twelve parallel lines to mimic trail pheromone trails (Fig. 2). Then, we waited four minutes to let the solvents evaporate before starting the behavioural trial so that spiders would be exposed to ant cues but not to the solvents. We started a behavioural trial by placing a spider in the middle of a test arena. This arena consisted of a Petri dish with one prepared filter paper and closed with a lid. All inner sides and walls of the Petri dish were covered with fluon to prevent the spiders from climbing and force them to stay on the filter paper. After the introduction, spiders exhibited typical exploration behaviour of the novel area after a short acclimation time. We then observed the spider after 5 min, 10 min, 3 h and 24 h and recorded which half of the filter paper the individual was resting on (blank or cue). We conducted several behavioural trials simultaneously by alternating arenas from both experiments (CHC, TP) and by alternating the orientation of the filter paper halves of both treatments (blank, cue) for randomisation purposes. After the behavioural trials, we released the spiders back to the fields where we had collected them.

**Table 2:** The body measurements and the number of individuals tested in hydrocarbon and pheromone experiments per species (*Pisaura mirabilis* and *Xysticus* spp.)

Parameter	Species	Cuticular hydrocarbon		Trail pheromone		F	df	*P*
		mean ± se	N	mean ± se	N			
Prosoma width (mm)	*P. mirabilis*	0.95 ± 0.02	54	0.96 ± 0.02	52	0.2	1,104	0.68
	*Xysticus*	0.77 ± 0.02	48	0.76 ± 0.02	50	0.1	1,96	0.76
Length (mm)	*P. mirabilis*	2.5 ± 0.06	54	2.5 ± 0.06	52	0.1	1,104	0.8
	*Xysticus*	1.6 ± 0.04	48	1.54 ± 0.04	50	0.3	1,96	0.58
Mass (mg)	*P. mirabilis*	2.2 ± 0.13	54	2.3 ± 0.13	52	0.3	1,104	0.62
	*Xysticus*	0.92 ± 0.08	48	0.96 ± 0.07	50	0.2	1,96	0.67

For each of the parameters and spider species, we fitted linear models [lm function in “stats” package (R Core Team 2023)] and validated results via permutation tests [100000 permutations; PermTest function in “pgirmess” package (Giraudoux 2023)]. There were no significant differences in all measured body parameters between experiments

### Statistical Analysis

Our response variable was the proportion of individuals recorded on the blank side of the filter paper. We first fitted an overall generalised linear mixed model (with a binomial error distribution (logit link) using “cue type” (CHC, TP), “time” (in minutes; continuous variable), “spider species” (*Pisaura mirabilis*, *Xysticus* spp.) and the interactions “cue type × time” and “cue type × species” as fixed predictors and “individual ID” as a random effect (see supplementary information section). Due to only significant differences based on “cue type” (*P* = 0.033) and the marginal difference between the tested species (*P* = 0.098), we then fitted separate models for each species and cue type. We used “time” as a continuous predictor (time in minutes) and “individual ID” as a random effect. In binomial models, a zero-value intercept means an equal probability of getting a response value of 1 (spider on the blank side) or 0 (spider on the cue side; Hosmer et al. 2013). Thus, we used the *P* value of the intercept to test the null hypothesis of a zero-value intercept, i.e., that spiders did not prefer the cue side over the blank side. We conducted the analyses in R (version 4.3.0; R Core Team 2023) through RStudio (version 2023.06.0; Posit team 2023) using the packages “lme4” (*glmer* function; Bates et al. 2015) and “car” (*Anova* function; Fox and Weisberg 2019) to obtain *P* values. Model diagnostics were performed using the package “DHARMa” (*simulateResiduals* and *testDispersion* functions; Hartig 2022). The figures supporting statistical analyses were created using the “ggplot2” (*ggplot*,* geom_point*,* geom_errorbar*,* ggsave*,* scale_color_manual*,* scale_fill_manual*,* ggtitle*,* theme_bw*,* theme*,* scale_y_continuous*,* labs and annotate* functions; Wickham 2016), “gridExtra” (*grid.arrange* function; Auguie 2017) and “viridis” (*viridis* function; Garnier et al. 2024) packages.

## Results

**Fig. 3:**
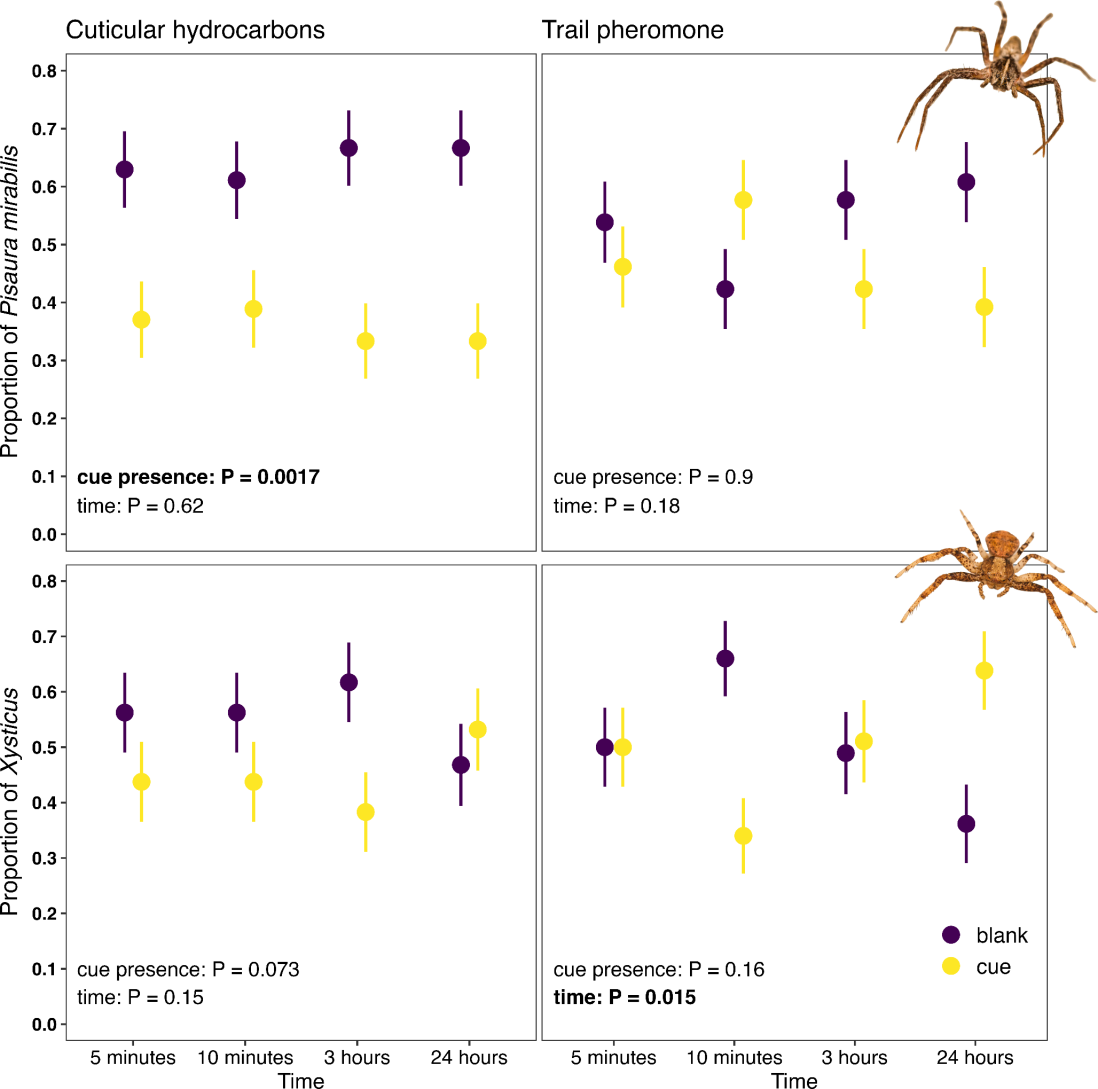
Effect of ant cues on site choice (cue vs. blank) of spiders depending on cue type (cuticular hydrocarbons, trail pheromone) and spider species (*Pisaura mirabilis*, *Xysticus* spp.). We used different sets of spiders for each cue type and measured their response at four time points. Mean proportions ± standard errors are presented. Spider pictures were taken by LM

*Pisaura mirabilis* avoided cuticular hydrocarbons. In the hydrocarbon experiment, the proportion of *Pisaura* individuals on the blank side was, on average, 1.8 times higher than on the cue side (on average, 64.5% and 35.5% on blank and cue, respectively; Wald *χ*^2^_1_ = 9.8, *P* = 0.0017; Fig. 3; Table 3). In contrast, these proportions did not differ in the trail pheromone experiment (on average, 53.8% and 46.2% on blank and cue, respectively; Wald *χ*^2^_1_ = 0.02, *P* = 0.9; Fig. 3; Table 3). The patterns were consistent over time both during hydrocarbon (Wald *χ*^2^_1_ = 0.24, *P* = 0.62) and trail pheromone experiments (Wald *χ*^2^_1_ = 1.8, *P* = 0.18).

**Table 3:** The number of individuals observed either on the blank or cue site for each time point in the hydrocarbon and the trail pheromone experiments per species (*Pisaura mirabilis* and *Xysticus* spp.)

Species	Time	Cuticular hydrocarbon		Trail pheromone	
		blank	cue	blank	cue
*P. mirabilis*	5 min	34	20	28	24
	10 min	33	21	22	30
	3 h	36	18	30	22
	24 h	36	18	31	20
*Xysticus*	5 min	27	21	25	25
	10 min	27	21	33	17
	3 h	29	18	23	24
	24 h	22	25	17	30

*Xysticus* spiders tended to avoid hydrocarbons as well, however, only with marginal significance. Spider proportions on blank sides tended to be higher than on the cue side (on average, 55.3% and 44.7% on blank and cue, respectively; Wald *χ*^2^_1_ = 3.2, *P* = 0.073; Fig. 3; Table 3). This was independent of time (Wald *χ*^2^_1_ = 2.1, *P* = 0.15; Fig. 3; Table 3). As in *P. mirabilis*, there was no main effect of the cue treatment on the choice of *Xysticus* in the trail pheromone experiment (on average, 50.3% and 49.7% on blank and cue, respectively; Wald χ^2^_1_ = 2.0, *P* = 0.16). However, there were significant temporal dynamics with around twice as many *Xysticus* individuals on blanks than cue-sites after 10 min but also twice as many individuals on cues than blank-sites after 24 h (Wald *χ*^2^_1_ = 5.9, *P* = 0.015; Fig. 3; Table 3).

## Discussion

Contrary to our expectations, *P. mirabilis* spiders only avoided long-lived (cuticular hydrocarbons) but not short-lived (trail pheromone) ant cues. The response of *Xysticus* was similar but weaker. As expected, the response to cuticular hydrocarbons did not weaken over 24 h after the presence of ants, which is in accordance with the low volatility of cuticular hydrocarbons.

### Volatile Versus Non-volatile Cues

Spiders mostly responded to long-lived cuticular hydrocarbons. The temporary avoidance of trail pheromones by *Xysticus* after ten minutes suggests that there could be a short-term effect, but the higher presence of *Xysticus* on cues after 24 h seems unexplainable: although at that time point trail pheromones had already evaporated entirely from the area, an equal distribution was expected (as after 3 h; Fig. 3), which is what our model with both spider species pooled (and thus higher statistical power) showed (Fig. S1 in supplementary information). Due to the higher volatility of trail pheromones compared to the cuticular hydrocarbons (Beckers et al. 1993; Evison et al. 2008; Lenoir et al. 2009), the spiders might not have evolved the ability even to detect fresh trail pheromones. The trail pheromones in the area might often be coupled with the immediate presence of predators, making the evolution of volatile semiochemicals’ detection superfluous due to the presence of visual and vibrational ant cues.

As highly complex mixtures of long-chained alkanes, *Lasius niger* cuticular hydrocarbons contain a mix of *n*-, monomethyl-, dimethyl-, and trimethyl- alkanes, and a smaller amount of alkenes (Blomquist 2010; Lenoir et al. 2009; Menzel et al. 2019; Wittke et al. 2022). Apart from intra-specific communication (Leonhardt et al. 2016; Sprenger and Menzel 2020), insect cuticular hydrocarbons primarily act as an external barrier preventing desiccation by restricting water loss (Blomquist and Bagnères 2010; Hadley 1994; Howard and Blomquist 2005), which can explain the persistent character of these semiochemicals. Spiders might hence develop the ability to detect the persistent cuticular hydrocarbons left by ants in the regularly patrolled area around the nests, but not the highly ephemeral trail pheromones. Since cuticular hydrocarbons are non-volatile, they accumulate in the area around the ant nest (“home-range marking”; Lenoir et al. 2009) and hence may signal enhanced ant activity in the area (Wüst and Menzel 2017). Although it is likely that even a single ant already poses a predation risk to a juvenile spider, a high density of hydrocarbons on the ground should indicate a high (and permanent) density of ants in the area. Hence, avoiding hydrocarbons should be more beneficial to spiders than avoiding trail pheromones.

### Different Responses of Tested Spider Species

Remarkably, in previous research, none of the two spider species changed its movement behaviour in the presence of ant cues in trials where full filter papers were covered within an enclosed arena (Mestre et al. 2020). In that study, significant changes in the movement were restricted to cursorial hunters. Both *P. mirabilis* and *Xysticus* are sedentary (sit-and-wait) predators predominantly as juveniles (Cardoso et al. 2011; Lenler-Eriksen 1969; Nyffeler & Breene 1990; Pekar et al. 2021). Moreover, another study by Mestre et al. (2014) demonstrated increased aerial dispersal in juvenile *Xysticus* spp. when exposed to areas previously enclosed with six alive *L. niger* individuals. Hence, *Xysticus* spp. has responded to the cues actively deposited by the *L. niger* in the area by aerial dispersal. Such mixed results from different experiments on the same study organism highlight the importance of an experimental setup that resembles natural conditions as closely as possible to obtain a more accurate response. For instance, *Xysticus* might have evolved the ability to detect the mixture of cues left by the walking ants in the area rather than to cuticular hydrocarbons or trail pheromones being presented in isolation and respond to the predation risk with their natural behaviour – dispersal using silk (Mestre et al. 2014). Hence, the application of single cue types and/or the lack of a possibility for aerial dispersal could be limitations to our experimental design that should be avoided in future experiments with *Xysticus* (also see Bucher et al. 2014; Bucher, Heinrich Bucher et al. 2015a, b for similar issues of different experimental designs). Nonetheless, our results with *P. mirabilis* spiders are straightforward, indicating their predator avoidance behaviour when confronted with ants’ cuticular hydrocarbons but not trail pheromone.

The response of *P. mirabilis* was stronger than that of *Xysticus*. It is unclear why *P. mirabilis* should avoid ants more strongly than *Xysticus*. Adult *P. mirabilis* may be more exposed to ants in their environment as they travel longer distances than *Xysticus*. However, the life stage that we tested, *P. mirabilis* were all juveniles known to build a sheet web for hunting right after abandoning the nursery web of their mothers (Lenler-Eriksen 1969). We could speculate that juvenile *P. mirabilis* should be protected from ants through their web. However, observations by Lenler-Eriksen (1969) indicate that juvenile *P. mirabilis* primarily uses its web for hunting and abandons it as soon as it is attacked. Also, the *P. mirabilis* individuals did not have time to produce a web during our experiments. In contrast, *Xysticus* individuals are ambush hunters known to hunt on the vegetation and on the ground with only infrequent movement (Nyffeler & Breene 1990), possibly reducing the risks of encountering ants compared to *P. mirabilis*. Nevertheless, ants are omnipresent (Schultheiss et al. 2022) and are known to also forage often on vegetation levels. Further, juvenile *Xysticus* are highly vagile and frequently disperse aerially from the elevated position on their silks when exposed to ants (Mestre et al. 2014). Thus, the weaker response of *Xysticus* than *P. mirabilis* to ant cuticular hydrocarbons in our experiments requires further investigation.

## Conclusion

Ants cuticular hydrocarbons caused avoidance behaviour in spiders, but the response to trail pheromones was unclear. The clear effect of hydrocarbons may be caused by their longer persistence compared to trail pheromones. Due to the co-occurrence of visual and vibrational ant cues simultaneously with ephemeral trail pheromones in nature, spiders might not evolve the ability to detect and avoid ants’ trail pheromones. Our study demonstrates that cuticular extracts from ants can be highly useful in the study of the ecology and evolution of avoidance behaviour in arthropods.

## Electronic Supplementary Material

Below is the link to the electronic supplementary material.

Supplementary Material 1

## Data Availability

Data generated and analysed during this study are available from Figshare: https://doi.org/10.6084/m9.figshare.25745928.v1.
