# Supplementary material for: Differential Effects of Ephemeral and Stable Predator Chemical Cues on Spider Antipredator Behaviour
Source: J Chem Ecol. 2024 Sep 21;50(11):714–24. doi: 10.1007/s10886-024-01543-5 (PMC11543770; doi:10.1007/s10886-024-01543-5)
Supplement: Supplementary file 1 — Supplementary Material 1 [file 10886_2024_1543_MOESM1_ESM.pdf]

Supplementary information

Differential effects of ephemeral and stable predator chemical cues on spider antipredator behaviour

Nijat Narimanov*, Jonna M. Heuschele, Martin H. Entling, Florian Menzel & Laia Mestre

Journal of Chemical Ecology

* Correspondence and present address: Nijat Narimanov; Institute of Organismic and Molecular Evolution (iomE), Johannes Gutenberg-University of Mainz, 55128 Mainz, Germany; Email: nnariman@uni-mainz.de / nijat.nariman@gmail.com;
ORCID: 0000-0003-1321-3243.

Results from the comprehensive model

The comprehensive model estimated significant differences based on cue type (hydrocarbon or trail pheromone; Wald χ^2_1 = 4.5, P = 0.033) and only marginal differences between studied species (*P. mirabilis* or *Xysticus*; Wald χ^2_1 = 2.7, P = 0.098), with no differences in time (Wald χ^2_1 = 0.9, P = 0.35) or interactions "cue type × time" (Wald χ^2_1 = 0.01, P = 0.92) and "cue type × species" (Wald χ^2_1 = 0.73, P = 0.39). We also analysed the data separately for each cue type (hydrocarbon and trail pheromone). In the hydrocarbon experiment, the proportion of individuals on the blank side was, on average, 1.5 times higher than on the cue side (Waldχ^2_1 = 14, P = 0.00016; Fig. S1), an effect that was consistent over time (Wald χ^2_1 = 0.35, P = 0.56) with only marginal differences between the spider species (Wald χ^2_1 = 3, P = 0.083). By contrast, there was no difference in the proportion of individuals choosing either side in the trail pheromone experiment (Wald χ^2_1 = 1.5, P = 0.23), and it depended neither on the time (Wald χ^2_1 = 0.53, P = 0.47) nor the species (Wald χ^2_1 = 0.36, P = 0.55).

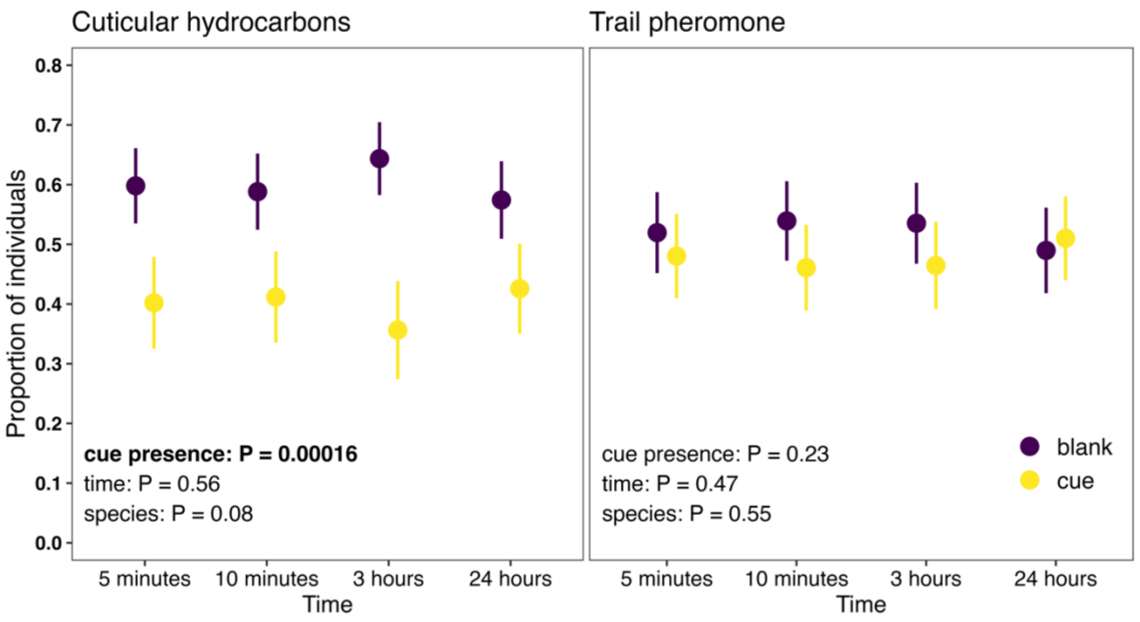

Fig. S1 Effect of cue type (cuticular hydrocarbons, trail pheromones) on site choice (cue vs. blank) for both spider species pooled (*Pisaura mirabilis*, *Xysticus* spp.). We used different sets of spiders for each cue type and measured their response at four time points. Mean proportions ± standard errors are presented.

Fig. S2 & S3

Web-link: https://figshare.com/s/a9a69654b481c3843dd6

DOI: 10.6084/m9.figshare.25744992

Testing the path choice accuracy of artificially prepared trail pheromone solution (dichloromethane + hindgut glands) on a Y-maze where we pipetted a path of trail pheromone solution on the left arm and dichloromethane alone on the other. Around 90% of the tested 30 workers successfully followed the path applied with the artificially prepared trail pheromone solution. Videos were filmed by LM and NN.
